# The Dual Role of Gastrodin in Spinal Cord Injury: Microglial Phenotype Switching and Neuronal Survival via PI3K/AKT Activation

**DOI:** 10.1002/cns.70811

**Published:** 2026-04-03

**Authors:** Jingsheng Feng, Shutao Gao, Yukun Hu, Weibin Sheng

**Affiliations:** ^1^ Department of Spinal Surgery The First Affiliated Hospital of Xinjiang Medical University Urumqi P. R. China

**Keywords:** gastrodin, microglial polarization, neuroinflammation, PI3K/AKT pathway, spinal cord injury

## Abstract

**Background:**

Spinal cord injury (SCI) triggers a complex secondary cascade, the defining feature of which is neuroinflammation. This amplifies tissue damage and impedes neurological recovery. Microglial polarization is a critical event in this process, yet effective modulation strategies remain limited.

**Objective:**

This study aimed to investigate whether gastrodin (GAS), a natural phenolic glycoside, could provide neuroprotection and promote functional recovery following SCI by modulating microglial polarization and to elucidate the underlying molecular mechanism.

**Methods:**

We employed a combination of behavioral, histological, and molecular assays, and a microglia–neuron co‐culture system under inflammatory conditions, using a rat contusion SCI model and LPS‐stimulated BV2 microglia in vitro. The role of the PI3K/AKT signaling pathway was specifically investigated using the inhibitor LY294002.

**Results:**

The administration of GAS markedly enhanced locomotor function, diminished lesion volume, and promoted neuronal survival in a dose‐dependent manner in vivo. GAS mitigated the inflammatory response by reducing M1 markers (iNOS and CD86) and augmenting M2 markers (Arg1 and CD206) within the injured spinal cord and BV2 microglia. Additionally, GAS exhibited a direct anti‐apoptotic effect on neurons in co‐culture. Mechanistically, GAS significantly activated the PI3K/AKT signaling pathway. Notably, the PI3K inhibitor LY294002 completely nullified the anti‐inflammatory and anti‐apoptotic effects of GAS, underscoring the central role of this pathway in mediating GAS's effects.

**Conclusion:**

This study demonstrates that GAS confers multifaceted protection against SCI by modulating microglial polarization from the pro‐inflammatory M1 phenotype to the anti‐inflammatory M2 phenotype and by directly inhibiting neuronal apoptosis, primarily through activation of the PI3K/AKT signaling pathway. These findings indicate that GAS holds significant potential as a therapeutic candidate for the treatment of SCI.

## Graphical abstract

Gastrodin promotes functional recovery after spinal cord injury by shifting microglial polarization from a damaging M1 to a protective M2 state and directly inhibiting neuronal apoptosis. This multi‐targeted neuroprotection is achieved primarily through activation of the PI3K/AKT signaling pathway, identifying gastrodin as a promising therapeutic candidate.

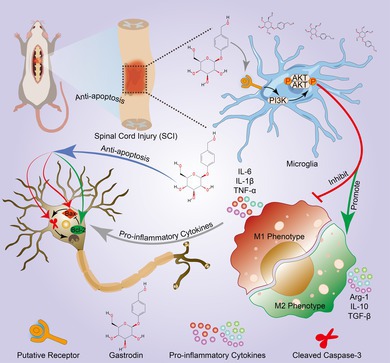

**Abbreviations**
AKT: protein kinase B
ANOVA: analysis of variance
Arg1: arginase‐1
BBB: Basso; Beattie; Bresnahan scale
BSA: bovine serum albumin
CCK‐8: cell counting kit‐8
DAPI: 4′,6‐diamidino‐2‐phenylindole
DMEM: Dulbecco's modified eagle medium
ELISA: enzyme‐linked immunosorbent assay
FBS: fetal bovine serum
GAS: gastrodin
H&E: hematoxylin and eosin
Iba1: ionized calcium‐binding adapter molecule 1
IF: immunofluorescence
IL‐10: interleukin‐10
IL‐1β: interleukin‐1 beta
iNOS: inducible nitric oxide synthase
LPS: lipopolysaccharide
NF‐κB: nuclear factor kappa‐B
PBS: phosphate‐buffered saline
PFA: paraformaldehyde
PI3K: phosphatidylinositol 3‐kinase
RT‐qPCR: quantitative reverse transcription polymerase chain reaction
SCI: spinal cord injury
SD: standard deviation
SPF: specific pathogen‐free
TLR4: toll‐like receptor 4
TNF‐α: tumor necrosis factor‐alpha
WB: western blot

## 1 Introduction

Spinal cord injury (SCI) is a severe and disabling neurological condition resulting in permanent sensory, motor, and autonomic dysfunction below the level of the injury [1]. Although the initial mechanical injury is irreversible, the subsequent secondary injury phase—characterized by vascular dysfunction, excitotoxicity, and persistent neuroinflammation—presents a critical window for therapeutic intervention [2]. Among the numerous secondary mechanisms, glial cell‐mediated neuroinflammation is recognized as a pivotal driver of injury expansion and a barrier to neural regeneration [3]. Activated microglia have been shown to polarize towards a pro‐inflammatory M1 phenotype, releasing toxic mediators such as tumor necrosis factor‐α (TNF‐α), interleukin‐1β (IL‐1β), and inducible nitric oxide synthase (iNOS). Alternatively, they can transition to an anti‐inflammatory M2 phenotype, secreting repair‐promoting factors like interleukin‐10 (IL‐10) and arginase‐1 (Arg‐1) [4]. In the context of aSCI, the microenvironment is characterized by the predominance of M1 polarization, a phenomenon that has been shown to exacerbate neuronal death and glial scar formation. Consequently, the guidance of microglia towards an M2 phenotype is regarded as a promising approach for mitigating secondary injury and promoting functional recovery [5, 6, 7].

The PI3K/AKT signaling pathway is a central axis that regulates cell survival, proliferation, metabolism, and inflammatory responses [2, 8]. Its protective role in neurological disorders has received significant attention. Research has indicated that the activation of the PI3K/AKT pathway following SCI has been shown to have a significant effect on the transduction of proinflammatory signals, such as NF‐κB, and to reduce M1 polarization of microglia, while promoting their transition to the M2 phenotype. This, in turn, has been demonstrated to improve the tissue repair environment [9]. The present study demonstrates that PI3K/AKT activation suppresses the TLR4/NF‐κB signaling pathway and fosters M2‐type microglial polarization. These findings underscore the significance of PI3K/AKT activation as a promising therapeutic target [10]. Despite the pathway's significant therapeutic potential, achieving precise and controllable in vivo regulation remains challenging due to issues such as drug delivery, spatiotemporal specificity, and safety.

Gastrodin (GAS), the primary active component extracted from the traditional Chinese medicinal herb Gastrodia elata, exhibits excellent blood–brain barrier permeability and safety. The substance has been demonstrated to possess significant neuroprotective, anti‐inflammatory, and antioxidant properties in a variety of neurological disease models, including cerebral ischemia, Alzheimer's disease, and Parkinson's disease [11, 12, 13]. However, systematic studies are lacking on whether it functions by modulating microglial polarization phenotypes in SCI and whether its specific molecular mechanisms depend on the PI3K/AKT pathway.

Based on the above background, this study proposes the scientific hypothesis that GAS may alleviate neuroinflammatory responses and inhibit neuronal apoptosis after SCI by activating the PI3K/AKT signaling pathway to regulate the phenotypic transition of microglia from M1 to M2, ultimately promoting neurological recovery. In order to validate this hypothesis, a rat spinal cord contusion model was established, and a comprehensive approach combining behavioral scoring, tissue staining, immunofluorescence, Western blot, flow cytometry, and cell co‐culture techniques was employed. This facilitated a systematic evaluation of GAS's effects on the inflammatory microenvironment, cellular polarization status, and key signaling pathways following SCI at both in vivo and in vitro levels. This study not only unveils a novel mechanism for GAS's neuroprotective effects but also provides theoretical and experimental support for its potential development as a targeted therapeutic agent for SCI.

## 2 Materials and Methods

### 2.1 Animals and Ethical Statement

Adult female Sprague–Dawley rats (8–10 weeks old, 200–220 g) were obtained from the Animal Experiment Center of Xinjiang Medical University. Animals were housed under standard specific pathogen‐free conditions (22°C ± 2°C, 50% ± 10% humidity, 12 h light/dark cycle) with ad libitum access to food and water. After at least 7 days of acclimatization, rats were randomly allocated to the experimental groups. Female rats were selected to facilitate post‐operative care (e.g., bladder management) and to minimize aggression‐related stress in group housing, thereby reducing potential confounding and animal loss after SCI. All experimental procedures were approved by the Animal Welfare and Research Ethics Committee of Xinjiang Medical University (Approval ID: 230306–110) and were conducted in strict accordance with the NIH Guide for the Care and Use of Laboratory Animals.

### 2.2 SCI Model and in Vivo GAS Administration

The rats were randomly separated into four groups, with each group having 12 members: (1) Sham group: subjected to laminectomy only; (2) SCI group: subjected to SCI and administered an equivalent volume of saline; (3) GAS‐50 group: subjected to SCI and administered 50 mg/kg GAS; (4) GAS‐100 group: subjected to SCI and administered 100 mg/kg GAS. Randomization was carried out with a sequence of random numbers generated by a computer.

The SCI model was implemented using a modified Allen's weight‐drop method while maintaining aseptic conditions. Briefly, rats were deeply sedated using 1% sodium pentobarbital (50 mg/kg, i.p.). A midline incision was made on the dorsal side, and the paravertebral muscles were carefully separated to expose the T9‐T10 vertebrae. A T10 laminectomy was performed to expose the dorsal surface of the spinal cord. A moderate contusion injury was induced by dropping a 10 g weight from a height of 6 cm onto the exposed dura mater. Successful model induction was confirmed by observing tail flicking and spasmodic hindlimb movements. The muscles and skin were then sutured in layers. Sham‐operated rats underwent the same surgical procedure except for the impact. Postoperatively, all SCI rats received antibiotics (cefuroxime sodium) for 3 days and manual bladder expression twice daily until spontaneous urination returned (approximately 7 days).

GAS (dissolved in saline) or an equivalent volume of saline was administered intraperitoneally (i.p.) twice daily (bid) starting immediately after surgery and continuing for 14 days. The doses of GAS (50 and 100 mg/kg) were chosen based on previous in vivo studies in rodents reporting neuroprotective/anti‐inflammatory efficacy and acceptable tolerability and to evaluate potential dose dependency [10, 14, 15].

### 2.3 Motor Function Evaluation

Hindlimb locomotor recovery was evaluated using the Basso–Beattie–Bresnahan (BBB) locomotor rating scale and the inclined plane test on postoperative days (POD) 1, 3, 7, 14, and 21, as outlined by Basso et al. [16] and Rivlin and Tator [17] [16, 17].

Behavioral assessments were conducted across all groups, each comprising 12 rats, where ’n’ represents the number of individual animals serving as biological replicates. All evaluations were performed by two independent observers who were blinded to the group allocations. For the BBB scoring, each rat was permitted to explore a circular open field, 1.2 m in diameter, for a duration of 4 min. Locomotor function was assessed based on standardized criteria, including hindlimb joint movement, trunk stability, interlimb coordination, paw placement, and weight support, utilizing a 21‐point scale (0 indicating no observable hindlimb movement and 21 indicating normal locomotion). The scores for the left and right hindlimbs were averaged to yield a single BBB score per animal at each time point, and the final score was determined as the mean of the ratings provided by the two observers.

In the inclined plane assessment, rats were oriented in a head‐down position on an adjustable platform with a rough surface. The angle of inclination was progressively increased from the horizontal at a constant rate of 5°per second. The maximum angle at which each rat could maintain its position for a minimum duration of 5 s without slipping was documented. Each rat participated in three consecutive trials, and the highest angle attained across these trials was utilized for further statistical analysis.

### 2.4 Tissue Collection and Processing

At designated time points after injury, rats were deeply anesthetized and transcardially perfused with 0.9% saline followed by 4% paraformaldehyde (PFA) in 0.1 M phosphate buffer (PB, pH 7.4). A 5‐mm segment of the spinal cord tissue centered on the lesion epicenter was dissected out. For histology and immunofluorescence, tissues were post‐fixed in 4% PFA overnight at 4°C, dehydrated in a graded sucrose series, embedded in paraffin, and sectioned coronally or sagittally at a thickness of 5 μm.

For protein analysis, a 2 cm segment of spinal cord surrounding the lesion site was rapidly dissected on ice. Tissues were homogenized in RIPA lysis buffer (PC101, Epizyme, Shanghai, China) containing 1% protease and phosphatase inhibitor cocktail (PR20015, Proteintech, Wuhan, China). The homogenates were centrifuged at 12,000 × ***g*** for 15 min at 4°C, and the supernatant was collected. Protein concentration was determined using a BCA protein assay kit (ZJ101, Epizyme, Shanghai, China).

### 2.5 Histological and Immunofluorescence Staining

For hematoxylin and eosin (H&E) staining, deparaffinized and rehydrated sections were stained with hematoxylin for 5 min, differentiated in 1% acid alcohol, blued in ammonia water, and counterstained with 0.5% eosin for 1 min. After dehydration and clearing, sections were mounted with neutral balsam and observed under a light microscope (Olympus, Tokyo, Japan).

For immunofluorescence (IF) staining, antigen retrieval was performed on tissue sections using EDTA antigen retrieval buffer (pH 8.0). Cells or tissue sections were blocked with 10% bovine serum albumin (BSA) for 1 h at room temperature and then incubated overnight at 4°C with the following primary antibodies: Arg‐1 (1:200, 16,001–1‐AP, Proteintech), iNOS (1:200, 22,226–1‐AP, Proteintech), Iba1 (1:200, GB12105‐50, Servicebio). After washing, samples were incubated with appropriate fluorescently labeled secondary antibodies (Cy3‐conjugated goat anti‐rabbit, GB21303, Servicebio; Alexa Fluor 488‐conjugated goat anti‐mouse, GB25301, Servicebio) for 1 h at room temperature in the dark. Nuclei were counterstained with DAPI. Images were captured using a Dmi8 fluorescence microscope (Leica, Germany).

### 2.6 Cell Culture and Treatments

The murine microglial cell line BV2 and the murine hippocampal neuronal cell line HT22 were obtained from the Cell Bank of the Chinese Academy of Sciences (Shanghai, China). BV2 cells were cultured in high‐glucose DMEM supplemented with 10% fetal bovine serum (FBS, Gibco) and 1% penicillin–streptomycin (P/S, HyClone). HT22 cells were maintained in DMEM/F12 medium with 10% FBS and 1% P/S. All cells were cultured at 37°C in a humidified incubator with 5% CO_2_.

To establish an inflammatory model in vitro, BV2 cells were seeded at an appropriate density and starved in serum‐free medium for 12 h upon reaching 70%–80% confluence. Cells were then pretreated with various concentrations of GAS (1 μg/mL, 10 μg/mL) or vehicle for 1 h, followed by stimulation with lipopolysaccharide (LPS, 1 μg/mL, L2880, Sigma‐Aldrich) for 24 h. Experimental groups were as follows: Control, LPS, LPS + GAS‐1 μg, LPS + GAS‐10 μg.

### 2.7 Co‐Culture Experiments

To explore the interactions between microglia and neurons and evaluate the neuron‐autonomous neuroprotective effects of GAS under conditions of microglia‐derived inflammatory stress, a non‐contact Transwell co‐culture system was developed utilizing inserts with 0.4 μm pores (Corning, Kennebunk, ME, USA). BV2 microglial cells were seeded in the upper chamber and cultured for 24 h, followed by activation with lipopolysaccharide (LPS) at a concentration of 1 μg/mL for an additional 24 h. Importantly, the BV2 cells were stimulated with LPS in the absence of GAS. Concurrently, HT22 neuronal cells were pretreated with GAS at concentrations of 1 or 10 μg/mL, or with a vehicle control, for 1 h, after which they were thoroughly washed with PBS and seeded in the lower chamber. Following the activation of BV2 cells, the inserts containing these cells were transferred to wells containing HT22 cells to initiate co‐culture, which was maintained for 24 h. Subsequently, cells from the upper (BV2) and lower (HT22) compartments were collected separately for Western blot analysis.

### 2.8 Microglia‐Conditioned Medium (CM) Transfer Experiments

To eliminate any potential confounding direct effects of GAS on neurons and to specifically evaluate the microglia‐mediated indirect neuroprotective effects, a BV2 CM transfer paradigm was employed. BV2 cells were pretreated with GAS at concentrations of 1 or 10 μg/mL, or with a vehicle control, for a duration of 1 h. Following pretreatment, the cells were washed twice with PBS and subsequently stimulated with LPS at a concentration of 1 μg/mL for 24 h in a GAS‐free medium. The resultant supernatants were collected as CM, centrifuged to remove cellular debris, and immediately transferred to HT22 neuronal cultures. It is important to note that HT22 neurons were not exposed to GAS at any stage of this experimental protocol. After a 24 h incubation period with the CM, HT22 cells were harvested for Western blot analysis to assess apoptosis‐related protein expression.

### 2.9 Flow Cytometry

BV2 cells were harvested by gentle scraping with cold PBS after treatments. Cells were washed and resuspended in staining buffer (PBS with 1% BSA). To block Fc receptors, cells were incubated with the anti‐CD16/32 antibody for 15 min on ice. Subsequently, cells were stained with fluorescently conjugated antibodies against CD86‐PE and CD206‐APC (or corresponding isotype controls) for 30 min at 4°C in the dark. After washing, cells were analyzed using a flow cytometer (Beckman, United States, dxflex). A minimum of 10,000 events per sample were acquired, and data were analyzed using Kaluza (2.0).

### 2.10 Western Blot Analysis

Equal amounts of protein lysates were separated by SDS‐PAGE and transferred onto PVDF membranes. The membranes were blocked with 5% non‐fat milk for 1 h at room temperature and then incubated overnight at 4°C with the following primary antibodies: β‐Actin (1:5000, 20,536–1‐AP, Proteintech), Arg‐1 (1:2000), iNOS (1:2000), Phospho‐PI3 Kinase p85 (Tyr467)/p55 (Tyr199) (1:1000, ab278545, Abcam), total PI3 Kinase p85 (1:4000, 60,225–1‐Ig, Proteintech), Phospho‐AKT (Ser473) (1:2000, 28,731–1‐AP, Proteintech), total AKT (1:5000, 10,176–2‐AP, Proteintech), Bcl‐2 (1:4000, 26,593–1‐AP, Proteintech), Bax (1:4000, 50,599–2‐Ig, Proteintech), Cleaved Caspase‐3 (1:1000, 9661 T, CST). After incubation with HRP‐conjugated secondary antibodies (1:5000, Proteintech) for 1 h at room temperature, protein bands were visualized using an ECL kit (Proteintech) and imaged with a ChemiDoc imaging system (Bio‐Rad). Band intensities were quantified using ImageJ software (NIH) and normalized to β‐Actin.

### 2.11 RNA Isolation and Real‐Time Quantitative Polymerase Chain Reaction (RT‐qPCR)

Total RNA was extracted using TRIzol reagent. cDNA was synthesized from 1 μg of RNA using a reverse transcription kit with gDNA eraser (Takara). qRT‐PCR was performed using TB Green Premix Ex Taq (Takara) on a real‐time PCR system. The thermocycling conditions were 95°C for 30 s, followed by 40 cycles of 95°C for 5 s and 60°C for 30 s. The 2^(–ΔΔCt) method was used to calculate relative mRNA expression levels, normalized to Gapdh. Primer sequences are listed in Table S1.

### 2.12 Enzyme‐Linked Immunosorbent Assay (ELISA)

The levels of inflammatory cytokines (IL‐1β, IL‐6, TNF‐α) in spinal cord homogenates or cell culture supernatants were measured using specific commercial ELISA kits (TNF‐α JONLNBIO JL13202; IL‐1β JONLNBIO JL20884; IL‐6 JONLNBIO JL20896; IL4 JONLNBIO JL20894‐96 T; IL‐10 JONLNBIO JL20242; TGF‐ β JONLNBIO JL13643) according to the manufacturers' instructions. Absorbance was read at 450 nm using a microplate reader.

### 2.13 Cell Viability Assay (CCK‐8)

Cell viability was assessed using the Cell Counting Kit‐8 (CCK‐8, Dojindo, Japan, catalogue no. CK04) in accordance with the manufacturer's instructions. In brief, cells were seeded into 96‐well plates at a density of 5 × 10^3^ cells per well in 100 μL of complete medium, after which they were allowed to adhere for 24 h. The culture medium was then replaced with fresh medium containing a vehicle control (PBS) or varying concentrations of GAS (0.1, 0.5, 1, 5, 10, 50, 100 and 500 μg). After 24 h of incubation, 10 μL of CCK‐8 solution was added to each well and the plates were incubated for a further 2 h at 37°C. Absorbance was measured at 450 nm using a microplate reader (SpectraMax iD5, Molecular Devices). Background absorbance from wells containing culture medium and CCK‐8 reagent without cells was subtracted. Cell viability was calculated as a percentage relative to the vehicle control group using the following formula:

$$\text{Cell viability}\;\left(\%\right)=\left[\left(\mathrm{As}–\mathrm{Ab}\right)/\left(\mathrm{Ac}–\mathrm{Ab}\right)\right]\times 100\%.$$

where As is the absorbance of the drug‐treated well, Ac is the average absorbance of the vehicle control wells, and Ab is the average background absorbance.

### 2.14 Statistical Analysis

Data are reported as mean ± standard deviation (SD) derived from a minimum of three independent experiments. Statistical analyses were conducted utilizing SPSS software version 26.0. Following verification of normality and homogeneity of variance, comparisons between two groups were assessed using the unpaired Student's *t*‐test. For comparisons among multiple groups, one‐way analysis of variance (ANOVA) was employed, followed by Tukey's post hoc test. A *p*‐value of less than 0.05 was deemed to indicate statistical significance.

## 3 Results

### 3.1 GAS Alleviates Secondary Injury and Improves Motor Function After SCI

**FIGURE 1:**
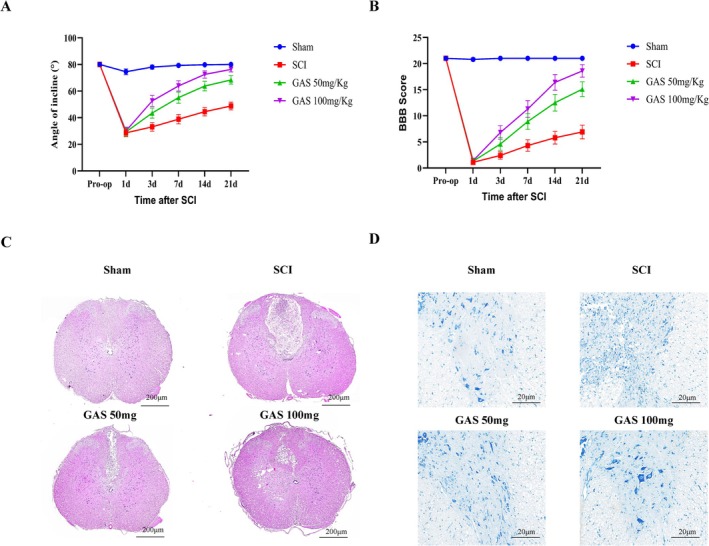
GAS improves pathological outcomes and promotes functional recovery after SCIs. (A, B) Inclined plane test results and Basso–Beattie–Bresnahan (BBB) locomotor scores of different groups over a 21‐day period post‐injury; *n* = 12 rats per group. (C) Representative images of H&E‐stained transverse sections of the spinal cord at 21 days post‐injury (dpi). Scale bar = 200 μm. (D) Representative images of Nissl‐stained transverse sections of the spinal cord at 21 dpi. Scale bar = 20 μm.

The extent of motor functional recovery serves as a pivotal indicator for assessing the prognosis of SCI. Recovery was evaluated using the Basso, Beattie, Bresnahan (BBB) locomotor rating scale and the inclined plane test. As illustrated in Figure 1A, treatment with GAS significantly improved motor function in rats with SCI, exhibiting a dose‐dependent effect. To assess the therapeutic efficacy of GAS, spinal cord tissue samples were collected on the seventh day post‐injury. Histopathological changes were examined using hematoxylin and eosin (H&E) staining. Histological analysis revealed a reduction in the lesion area and improved preservation of tissue architecture in GAS‐treated groups (refer to Figure 1C). The high‐dose group (GAS‐100 mg/kg) exhibited the most pronounced effects. Additionally, Nissl staining indicated that GAS significantly increased the number of surviving neurons and decreased the number of apoptotic neurons, with the most substantial effects observed in the high‐dose group (Figure 1C).

### 3.2 GAS Attenuates Early Inflammatory Response and Modulates Microglial Polarization After SCI

**FIGURE 2:**
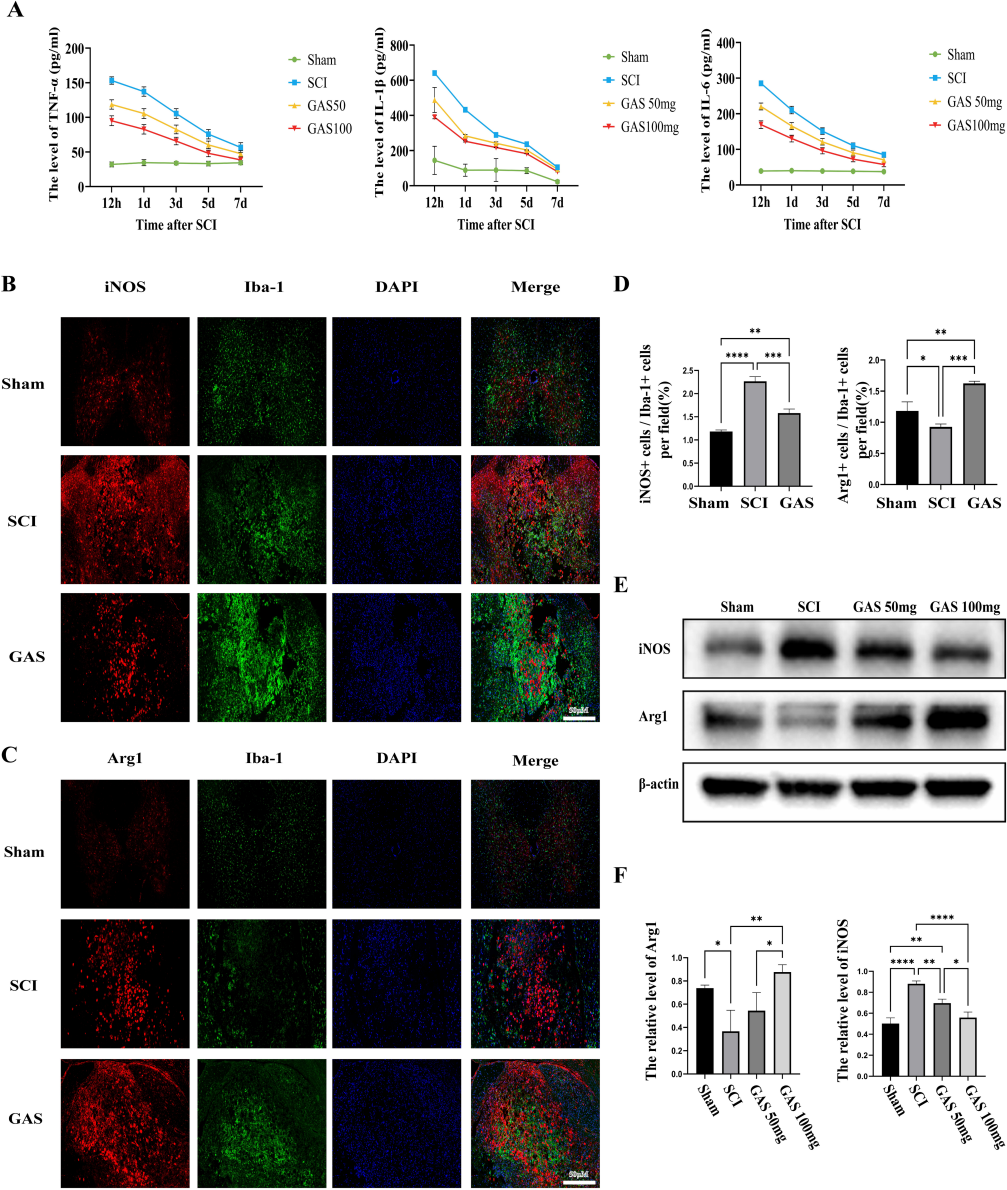
GAS attenuates the early inflammatory response and modulates microglial activation after SCI. (A) Concentration–time curves of pro‐inflammatory cytokines (TNF‐α, IL‐1β, IL‐6) in spinal cord homogenates from different groups of rats at various time points after SCI. (B) Representative co‐immunofluorescence images showing Iba1 (ionized calcium‐binding adapter molecule 1)‐positive microglia (green) expressing the pro‐inflammatory marker iNOS (red) in the lesion epicenter at 7 dpi. Scale bar = 50 μm. (C) Representative co‐immunofluorescence images showing Iba1‐positive microglia (green) expressing the anti‐inflammatory marker Arg1 (red) in the lesion epicenter at 7 dpi. Scale bar = 50 μm. (D) Quantitative analysis of the percentage of iNOS^+^Iba1^+^ and Arg1^+^Iba1^+^ cells among total Iba1^+^ microglia obtained from three regions of interest (ROIs). Data are mean ± SD; **p < 0.01 by Student's t‐test; *n* = 3 biological replicates. (E) Representative Western blots showing protein levels of iNOS and Arg1 in spinal cord tissues at 7 dpi. (F) Densitometric quantification of protein levels from (E). Data are mean ± SD; *p < 0.05, **p < 0.01, ***p < 0.001, ****p < 0.0001 by one‐way ANOVA; *n* = 3 rats per group.

Secondary injury following SCI, particularly neuroinflammation, significantly impacts neuronal survival. The levels of key inflammatory cytokines in spinal cord homogenates were measured using ELISA at various time points (12 h, and 1, 3, 5, and 7 days) post‐SCI. The analysis reveals that GAS treatment effectively mitigates the initial increase in proinflammatory factors and enhances the secretion of anti‐inflammatory factors (see Figure 2A and Figure S2). On the seventh day post‐injury, we conducted immunofluorescence co‐staining analyses to evaluate the polarization state of microglia, utilizing Iba‐1 in conjunction with either iNOS or Arg1. Iba‐1 (ionized calcium‐binding adapter molecule 1) functions as a microglia‐specific marker, with iNOS serving as an indicator of M1 polarization and Arg1 as an indicator of M2 polarization. The results indicated that, in comparison to the SCI group, the proportion of Iba‐1/iNOS double‐positive cells in the lesion center was significantly decreased following GAS treatment (Figure 2B,D). Conversely, the proportion of Iba‐1/Arg1 double‐positive cells was significantly increased (Figure 2C,D). Subsequent Western blot analysis revealed that GAS treatment led to a reduction in iNOS protein expression and an upregulation of Arg1 expression, with these effects being more pronounced in the high‐dose group (Figure 2E,F). Collectively, these findings suggest that GAS inhibits the pro‐inflammatory (M1) polarization of microglia and facilitates their transition to an anti‐inflammatory (M2) phenotype, thereby mitigating inflammatory responses subsequent to SCI.

### 3.3 GAS Suppresses LPS‐Induced Pro‐Inflammatory Activation of Microglia in Vitro

**FIGURE 3:**
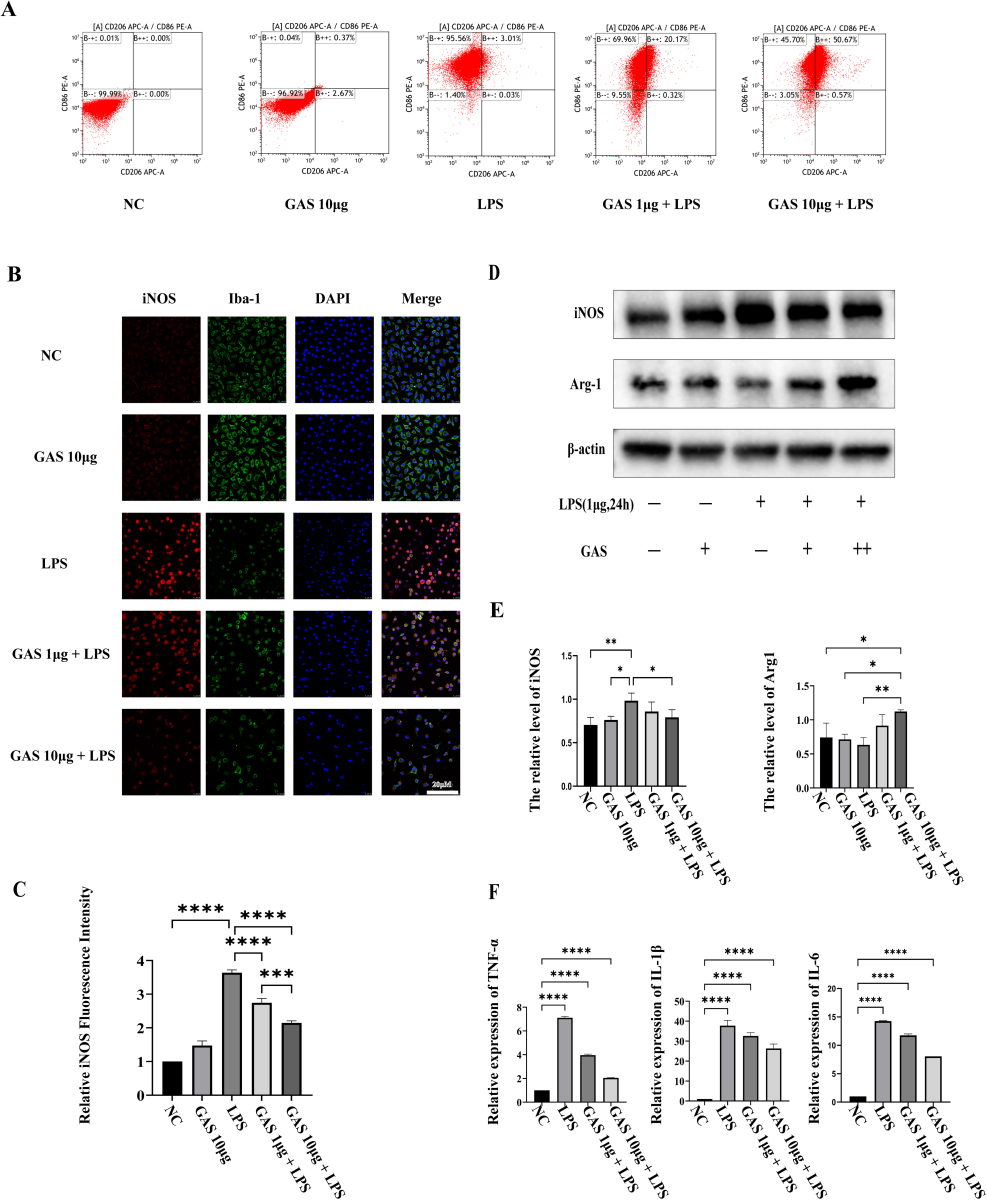
GAS suppresses LPS‐induced pro‐inflammatory activation of microglia in vitro. (A) Representative flow cytometry plots showing the polarization status of BV2 cells upon LPS induction and GAS treatment. (B) Representative immunofluorescence images of BV2 cells stained for Iba1 (green) and iNOS (red). Nuclei are counterstained with DAPI (blue). Scale bar = 20 μm. (C) Quantitative analysis of iNOS fluorescence intensity. Data are mean ± SD; *p < 0.05, **p < 0.01, ***p < 0.001, ****p < 0.0001 by Student's t‐test; data are pooled from three independent experiments. (D) Representative Western blots showing iNOS protein expression in BV2 cells. (E) Densitometric quantification of iNOS levels from (D). Data are mean ± SD; *p < 0.05, **p < 0.01 by one‐way ANOVA; data are from three independent experiments. (F) mRNA expression levels of pro‐inflammatory genes in BV2 cells measured by RT‐qPCR. All data were normalized to Gapdh expression.

To further explore the influence of GAS on microglial polarization, we examined its effects on LPS‐stimulated BV2 microglial cells. A CCK‐8 assay was performed to assess the cytotoxicity of GAS, revealing no significant toxicity at concentrations up to 500 μg (refer to Figure S1). Flow cytometry analysis indicated that GAS pretreatment significantly reduced the percentage of CD86‐positive (M1) cells while increasing the percentage of CD206‐positive (M2) cells in a concentration‐dependent manner (Figure 3A). Consistent with these findings, immunofluorescence staining showed that GAS inhibited LPS‐induced iNOS expression (see Figure 3B,C). Western blot analysis further confirmed that GAS downregulated iNOS and upregulated Arg1 protein levels (Figure 3D,E). Additionally, qPCR results demonstrated that GAS pretreatment reversed the LPS‐induced transcriptional upregulation of pro‐inflammatory marker genes (Figure 3F).

### 3.4 GAS Inhibits Neuronal Apoptosis After SCI and Protects Neurons Against Activated Microglia in Vitro

**FIGURE 4:**
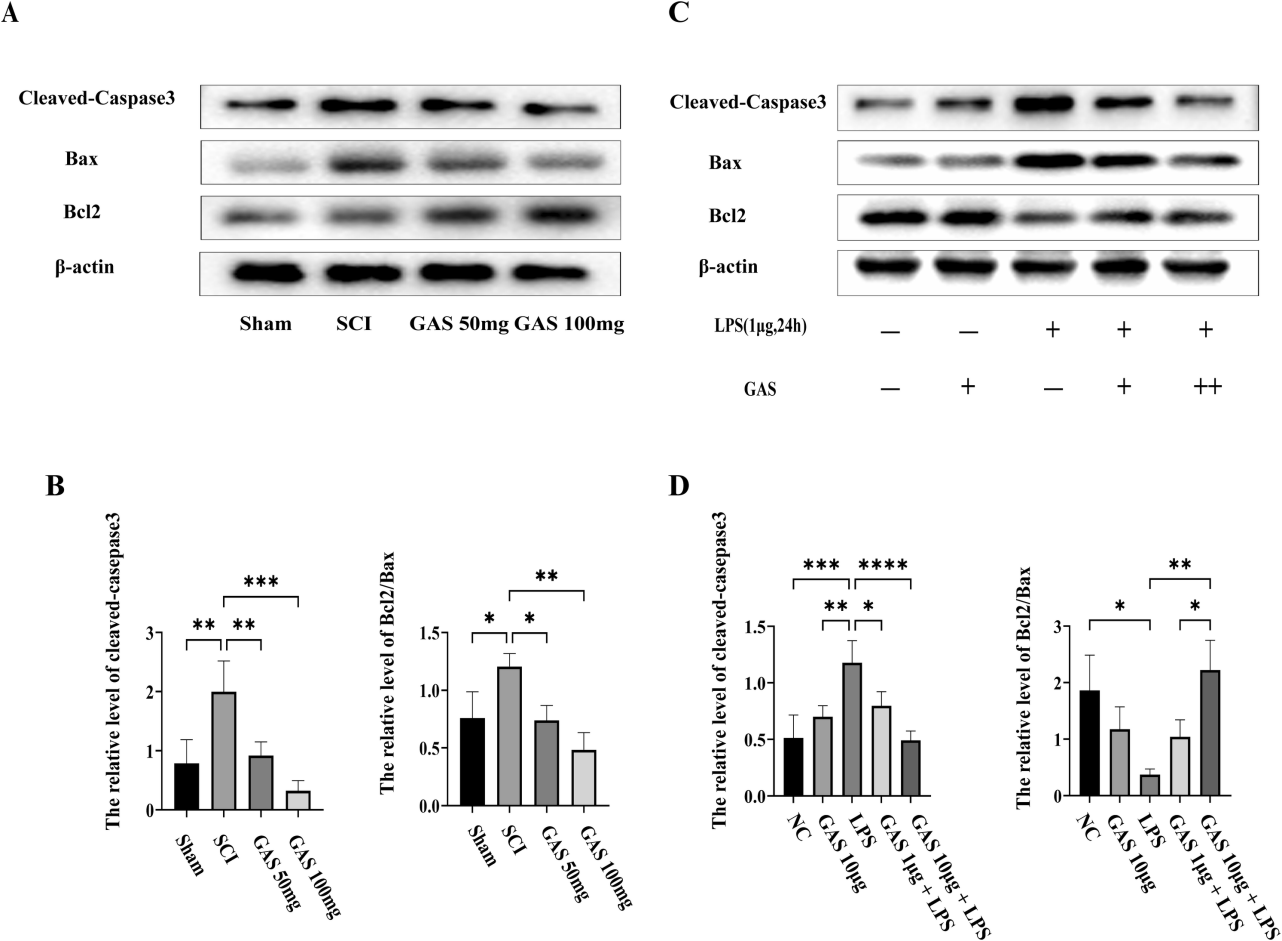
GAS inhibits neuronal apoptosis after SCI and directly protects neurons against activated microglia in vitro. (A) Representative Western blots showing the expression of Cleaved‐caspase‐3, Bcl‐2, and Bax in spinal cord tissues from different groups of rats after SCI. (B) Densitometric quantification of protein levels from (A). Data are mean ± SD; *p < 0.05, **p < 0.01, ***p < 0.001, ****p < 0.0001 by one‐way ANOVA; data are from three independent experiments. (C) Representative Western blots showing the expression of Cleaved‐caspase‐3, Bcl‐2, and Bax in HT22 neurons co‐cultured with differently treated BV2 microglia. (D) Densitometric quantification of protein levels from (C).

To evaluate the impact of GAS on neuronal apoptosis, Western blot analysis was performed to examine the expression of apoptosis‐related proteins in spinal cord tissues. GAS treatment markedly reversed the elevated levels of cleaved caspase‐3 observed under pathological conditions and significantly increased the Bcl‐2/Bax ratio (Figure 4A,B), indicating a robust anti‐apoptotic effect.

Using a non‐contact Transwell co‐culture system, in which GAS exposure was restricted to the neuronal compartment, GAS pretreatment similarly attenuated apoptosis‐related protein alterations in HT22 neurons exposed to LPS‐activated BV2 microglia. This neuroprotective effect occurred in a dose‐dependent manner, as evidenced by reduced cleaved caspase‐3 expression and an increased Bcl‐2/Bax ratio (Figure 4C,D).

To rigorously assess whether the observed neuroprotective effects were exclusively due to a direct interaction of GAS with neurons, we conducted a CM transfer experiment utilizing BV2 microglial cells. BV2 cells were pretreated with either GAS or a vehicle control, followed by stimulation with LPS. The resultant CM was collected and subsequently applied to HT22 neurons, ensuring that the HT22 cells were never directly exposed to GAS. CM obtained from LPS‐activated BV2 cells significantly increased pro‐apoptotic signaling in HT22 neurons. In contrast, CM from BV2 cells co‐treated with LPS and GAS substantially mitigated these apoptosis‐inducing effects (Figure S3A,B).

Collectively, these findings demonstrate that GAS confers neuroprotection under inflammatory stress through a dual mechanism involving both microglia‐mediated indirect effects and direct anti‐apoptotic actions on neurons.

### 3.5 GAS Treatment Activates the PI3K/AKT Signaling Pathway in the Injured Spinal Cord and in Microglia

**FIGURE 5:**
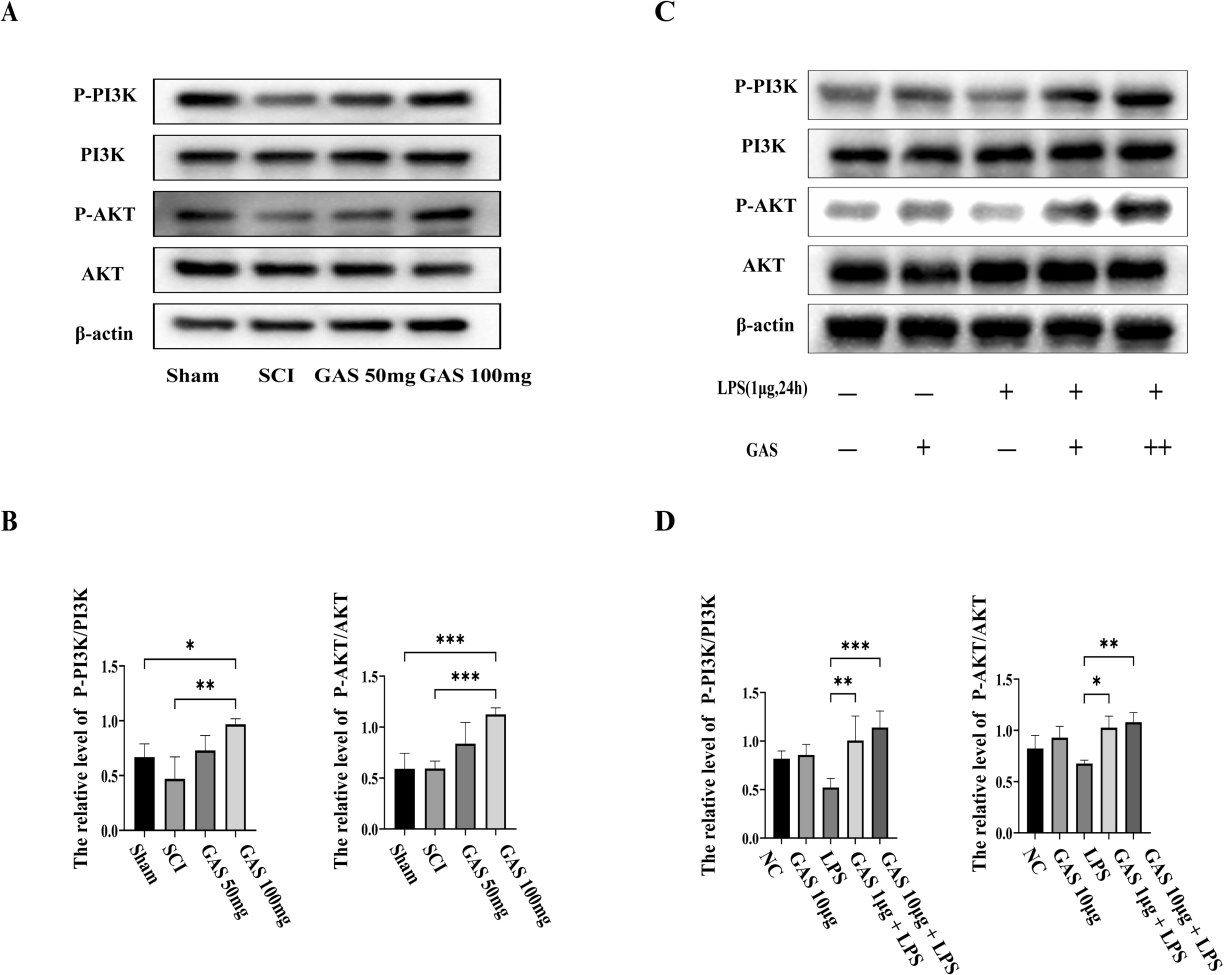
GAS treatment activates the PI3K/AKT signaling pathway in the injured spinal cord and in microglia. (A) Representative Western blots showing the expression of P‐PI3K, PI3K, P‐AKT, and AKT in spinal cord tissues from different groups after SCI. (B) Densitometric quantification of protein levels from (B). Data are mean ± SD; *p < 0.05, **p < 0.01, ***p < 0.001, ****p < 0.0001 by one‐way ANOVA; data are from three independent experiments. (C) Representative Western blots showing the expression of P‐PI3K, PI3K, P‐AKT, and AKT in BV2 cells under different treatments. (D) Densitometric quantification of protein levels from (C).

The PI3K/AKT signaling pathway has been implicated in the secondary injury mechanisms following SCI. Initial investigations into this process were performed using the spinal cord tissue, where western blot analysis demonstrated that the protein levels of phosphorylated PI3K (p‐PI3K) and AKT (p‐AKT) were significantly elevated in groups treated with GAS compared to both the sham and SCI groups, displaying a dose‐dependent response (refer to Figure 5A,B). Similar findings were observed in vitro, where GAS treatment led to increased levels of p‐PI3K and p‐AKT in BV2 cells under both basal conditions and those stimulated with LPS (refer to Figure 5C,D). These results suggest that GAS activates the PI3K/AKT pathway, potentially mediating its effects on microglial polarization and anti‐apoptotic processes.

### 3.6 PI3K/AKT Pathway Inhibitor LY294002 Reverses the Anti‐Inflammatory and Anti‐Apoptotic Effects of GAS

**FIGURE 6:**
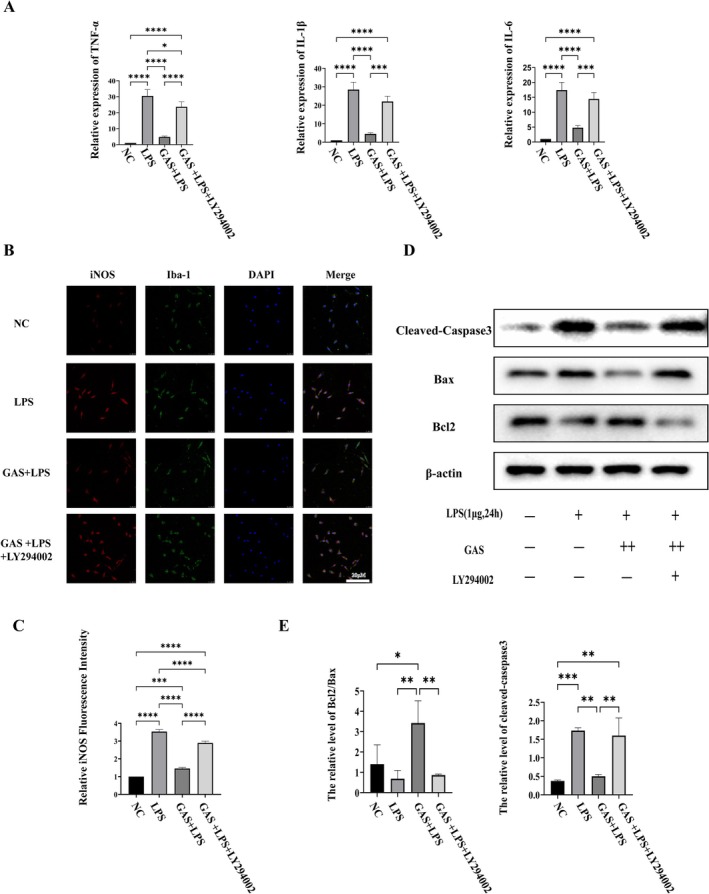
PI3K/AKT pathway inhibitor LY294002 reverses the anti‐inflammatory and anti‐apoptotic effects of GAS. (A) mRNA expression levels of pro‐inflammatory genes in BV2 cells measured by RT‐qPCR. All data were normalized to GAPDH expression. (B) Representative immunofluorescence images of BV2 cells stained for Iba1 (green) and iNOS (red). Nuclei are counterstained with DAPI (blue). Scale bar = 20 μm. (C) Quantitative analysis of iNOS fluorescence intensity. (D) Representative Western blots showing the expression of Cleaved‐caspase‐3, Bcl‐2, and Bax in HT22 neurons co‐cultured with BV2 microglia under different treatments. (E) Densitometric quantification of protein levels from (D). Data are mean ± SD; *p < 0.05, **p < 0.01, ***p < 0.001, ****p < 0.0001 by Student's t‐test; data are pooled from three independent experiments.

To determine whether the beneficial effects of GAS are dependent on the PI3K/AKT pathway, the specific PI3K inhibitor LY294002 was employed. Quantitative PCR analysis indicated that cotreatment with LY294002 resulted in increased mRNA expression levels of IL‐1β, IL‐6, and TNF‐α, thereby negating the anti‐inflammatory effects of GAS (Figure 6A). Immunofluorescence staining for iNOS revealed that LY294002 treatment increased the proportion of iNOS‐positive cells, thus reversing the GAS‐induced inhibition of M1 macrophage polarization (refer to Figure 6B,C). Additionally, Western blot analysis demonstrated that LY294002 nullified the GAS‐induced decrease in cleaved‐caspase‐3 and the elevation in the Bcl‐2/Bax ratio (Figure 6D,E). These findings strongly indicate that the PI3K/AKT pathway is essential for the anti‐inflammatory and anti‐apoptotic effects of GAS.

## 4 Discussion

The current study systematically elucidates a novel mechanism by which GAS exerts neuroprotective effects following SCI, integrating evidence from both in vivo and in vitro experiments. The primary finding is that GAS activates the PI3K/AKT signaling pathway, facilitating a shift in microglial polarization from the pro‐inflammatory M1 phenotype to the anti‐inflammatory M2 phenotype, while concurrently enhancing neuronal resistance to apoptosis. This signaling cascade has been demonstrated to mitigate secondary injury and promote motor function recovery. These findings not only highlight GAS as a promising multi‐target therapeutic candidate but also underscore the PI3K/AKT pathway as the central molecular switch mediating its effects. The integrative nature of this mechanism reveals the simultaneous targeting of both immune cells (microglia) and parenchymal cells (neurons) by GAS, thereby achieving synergistic therapeutic efficacy. This provides a critical theoretical foundation for the development of precise interventions targeting the neuroimmune microenvironment in SCI.

Functional motor recovery is the primary objective in the treatment of SCI, with the preservation of tissue architecture being fundamental to this process [18]. Initial evidence indicates that treatment with GAS significantly enhances BBB and LSS scores in rats while simultaneously reducing lesion volume and neuronal loss in a dose‐dependent manner (Figure 1). This concurrent improvement in both functional and structural outcomes strongly implies that GAS effectively disrupts the secondary injury cascade following SCI. A critical aspect of this cascade is microglia‐mediated neuroinflammation. The findings of the current study reveal that GAS treatment reduces systemic levels of pro‐inflammatory cytokines in vivo and, more importantly, rebalances microglial polarization by increasing the population of Arg1‐positive M2 cells and decreasing iNOS‐positive M1 cells at the lesion epicenter (see Figure 2). This result is significant because broad immunosuppression could potentially impair essential immune surveillance, whereas a strategy focused on polarization shift aims to transform a harmful inflammatory environment into a reparative one. The in vitro experiments further corroborated that GAS exerts a direct and concentration‐dependent effect on BV2 cell polarization (Figure 3), thereby excluding indirect influences mediated by other systemic factors in vivo. This observation indicates that the modulation of microglial phenotypic switching constitutes a pivotal upstream event in GAS‐mediated neuroprotection. This conclusion aligns with the research by Zuo Hanjun et al., which demonstrated that GAS attenuates microglia‐mediated inflammation through the PI3K/AKT pathway in a neonatal rat model of hypoxic–ischemic brain injury [15]. Collectively, these findings suggest the potential universality of GAS's anti‐inflammatory mechanism across various models of central nervous system injury.

An especially noteworthy discovery is that the protective effects of GAS extend beyond its role in immunomodulation. Numerous studies conducted in various neurological contexts have demonstrated that GAS exhibits significant anti‐apoptotic properties [19, 20]. In vivo analyses have shown that GAS treatment results in the suppressed expression of cleaved‐caspase‐3 and a marked increase in the Bcl‐2/Bax ratio (Figure 4A,B). Furthermore, co‐culture experiments have indicated that GAS not only indirectly safeguards neurons by “reprogramming” activated microglia but also directly enhances the anti‐apoptotic capacity of neurons in inflammatory conditions (Figure 4C,D). This dual mechanism, which concurrently targets both immune cells (microglia) and parenchymal cells (neurons), substantially enhances its therapeutic efficacy. This finding implies that GAS influences a critical signaling node common to both immune responses and apoptotic pathways [21].

Building on these findings, the research concentrated on the PI3K/AKT pathway, a well‐established master regulator of cell survival, proliferation, and inflammation [2, 22, 23]. A previous network pharmacology study also anticipated the involvement of the PI3K/AKT pathway in the action of GAS [24]. The current study reveals that GAS significantly enhances the phosphorylation levels of PI3K and AKT in both injured spinal cord tissues and LPS‐stimulated microglia (Figure 5), thereby providing a unified molecular basis for its diverse effects. Mechanistically, activated AKT promotes neuronal survival by phosphorylating and inhibiting pro‐apoptotic transcription factors such as GSK‐3β and FoxO [25, 26, 27]. Simultaneously, it has been shown to exert a negative regulatory effect on pro‐inflammatory signaling pathways such as NF‐κB, thereby suppressing M1 polarization and promoting M2 polarization [10, 28]. Additionally, the PI3K/AKT pathway may influence autophagy and oxidative stress by modulating downstream effectors such as mTOR or Nrf2, forming a complex protective network [29, 30, 31, 32].

While correlation does not necessarily indicate causation, the most compelling evidence in our study is derived from gain‐and‐loss experiments utilizing the specific PI3K inhibitor LY294002. The results demonstrated that inhibition of the PI3K/AKT pathway nearly completely negated all beneficial effects of GAS on inflammation (Figure 6A–C) and apoptosis (Figure 6D,E). This finding underscores the critical role of the PI3K/AKT pathway in the mechanism of action of GAS, thereby affirming that its involvement extends beyond a mere correlative relationship. The pathway functions as a “master switch” regulating the downstream protective events initiated by GAS. This suggests that future strategies focused on the precise modulation of this pathway could be crucial for optimizing the efficacy of GAS or even for the development of novel therapeutic interventions.

The theoretical significance of this study is rooted in its elucidation of a comprehensive signaling pathway, extending from GAS administration to the activation of the PI3K/AKT pathway, culminating in the polarization shift of microglia and the inhibition of neuronal apoptosis. This provides a detailed mechanistic framework for understanding the actions of this natural compound. From a translational standpoint, considering that GAS is an active constituent of a clinically utilized herb with an established safety profile, our study offers substantial preclinical evidence supporting its potential repurposing as a therapeutic agent for SCI treatment.

Despite the contributions of the present study, it is not devoid of limitations. First, although the central role of the PI3K/AKT pathway has been corroborated, further research is necessary to ascertain whether GAS directly interacts with PI3K or exerts its effects indirectly through upstream receptors, such as G‐protein‐coupled receptors (GPCRs) or growth factor receptors, to activate this pathway. Employing techniques such as molecular docking and surface plasmon resonance (SPR) to verify direct binding between GAS and potential targets, including PI3K, will constitute a crucial aspect of future research initiatives. Second, the lack of cell‐type‐specific gene knockout models, such as the use of CX3CR1‐Cre mice to selectively delete PI3K in microglia, constrains the ability to accurately delineate the relative contributions of this pathway in various cell types. Additionally, the long‐term effects of GAS on astrocytic scar formation and axonal regeneration warrant further investigation.

## 5 Conclusions

In conclusion, this study provides evidence that GAS significantly enhances functional recovery and reduces tissue damage following SCI in rats. The neuroprotective effects observed are primarily attributed to a marked reduction in neuroinflammation and the promotion of beneficial microglial polarization towards the M2 phenotype. Crucially, the study identifies the activation of the PI3K/AKT signaling pathway as the central mechanism underlying GAS's effects, as demonstrated by the reversal of its benefits upon PI3K inhibition. Despite these promising findings, further research is necessary to assess the clinical translational potential of GAS. Future studies should include investigations in large animal models and comprehensive toxicological evaluations. This research adds to the expanding body of literature supporting the therapeutic potential of targeting microglial polarization and the PI3K/AKT pathway in the treatment of SCI.

## Author Contributions

Jingsheng Feng designed this study and carried out most of the experimental work. Yukun Hu participated in data collection and validation. Jingsheng Feng drafted the manuscript. Shutao Gao and Weibin Sheng revised and approved the final version of the manuscript. All authors read and approved the submitted version.

## Funding

The National Natural Science Foundation of China (82360257); Xinjiang Uygur Autonomous Region* science and technology, innovation leading talent project, China·(2023TSYCLJ0031); Natural Science Foundation of Xinjiang Uygur Autonomous Region (2021D01D18).

## Conflicts of Interest

The authors declare no conflicts of interest.

## Supporting information


**Figure S1:** Dose–response effect of GAS on the viability of microglial cells. (A) Microglial cells were treated with increasing concentrations (0.1–500 μg/mL) of GAS for 24 h, and cell viability was assessed by the CCK‐8 assay. Note that even at the high concentration of 100 μg/mL, cell viability remained above 90%, indicating no significant cytotoxicity.

**Figure S2:** Gastrodin reshapes the cytokine milieu after spinal cord injury. (A) Concentration**–**time curves of anti‐inflammatory cytokines (IL‐10, TGF‐β, IL‐4) in spinal cord homogenates from different groups of rats at various time points after SCI.
(B) ELISA quantification of pro‐ and anti‐inflammatory cytokines (TNF‐α, IL‐1β, IL‐6, IL‐10, TGF‐β, and IL‐4) in spinal cord homogenates from the indicated groups at 12 h, 1 day, 3 days, 5 days, 7 days.

**Figure S3:** Conditioned medium from LPS‐stimulated BV2 cells induces apoptosis, which is attenuated by gastrodin. (A) Representative Western blot images showing the expression of cleaved caspase‐3, Bax, and Bcl‐2 in cells treated with normal control medium (NC), conditioned medium (CM), or CM supplemented with gastrodin (CM + GAS). β‐actin served as the loading control. (B) Densitometric quantification of cleaved caspase‐3 (normalized to β‐actin) and the Bcl‐2/Bax ratio from (A). Data are presented as mean ± SD from *n* = 3 independent experiments. Statistical significance was assessed using one‐way ANOVA followed by Tukey's post hoc test. *p < 0.05, **p < 0.01, ***p < 0.001.

**Table S1:** Primer sequences and expected amplicon sizes used for mouse RT–qPCR. Primer sequences are listed in the 5′→3′ direction. F and R denote forward and reverse primers, respectively. Product size indicates the expected RT–qPCR amplicon length (bp). Target genes included rat IL‐1β, IL‐6, and TNF‐α; GAPDH was used as the internal reference gene.

## Data Availability

All data supporting the findings of this study are included in this published article and its supplementary information files. Additional information is available from the corresponding author upon reasonable request.
