# Supplementary figures and images for: The Dual Role of Gastrodin in Spinal Cord Injury: Microglial Phenotype Switching and Neuronal Survival via PI3K/AKT Activation
Source: CNS Neurosci Ther. 2026 Apr 3;32(4):e70811. doi: 10.1002/cns.70811 (PMC13052207; doi:10.1002/cns.70811)

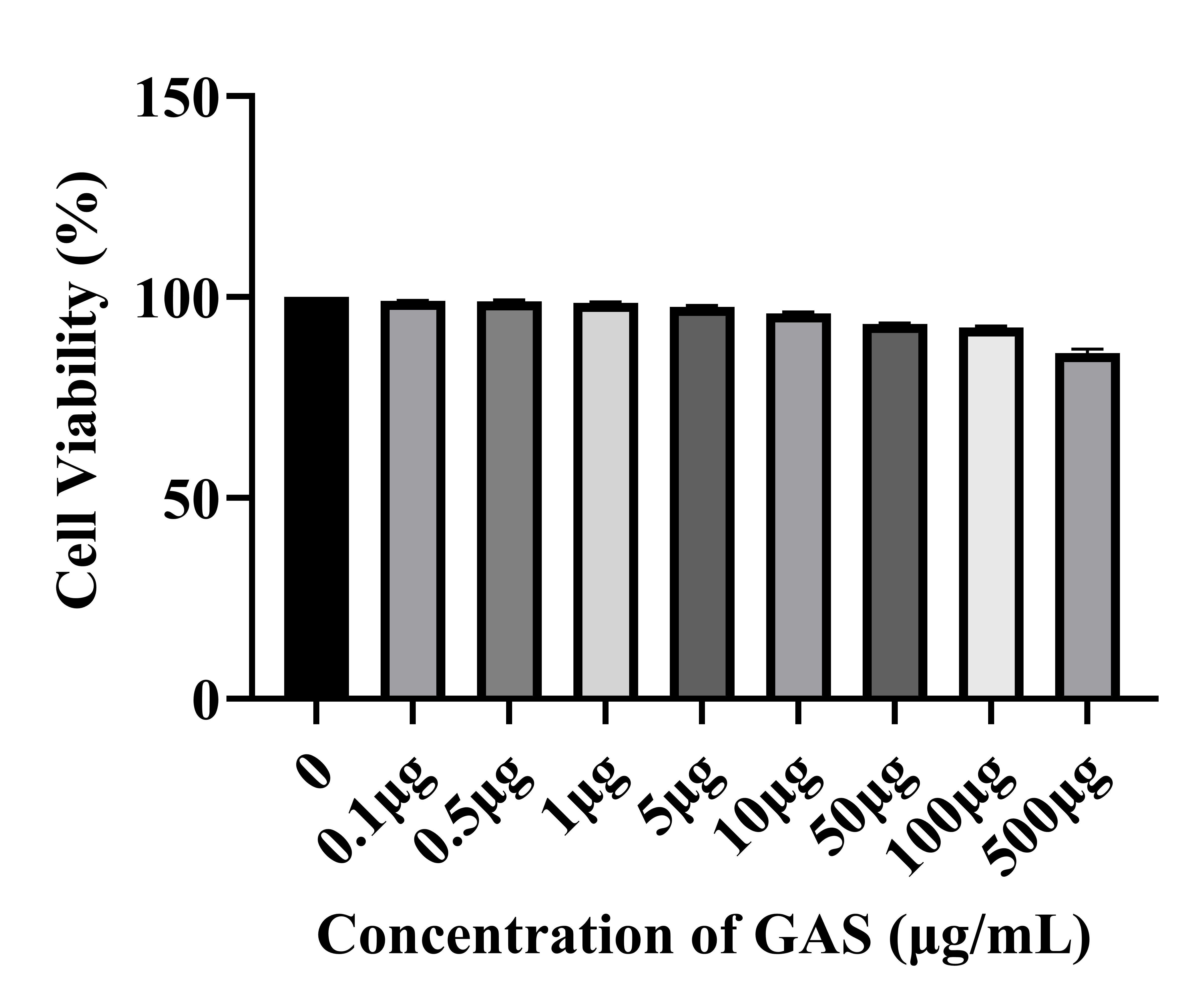

Supplement: Supplementary file 1 — Figure S1: Dose–response effect of GAS on the viability of microglial cells. (A) Microglial cells were treated with increasing concentrations (0.1–500 μg/mL) of GAS for 24 h, and cell viability was assessed by the CCK‐8 assay. Note that even at the high concentration of 100 μg/mL, cell viability remained above 90%, indicating no significant cytotoxicity. [file CNS-32-e70811-s004.tif]

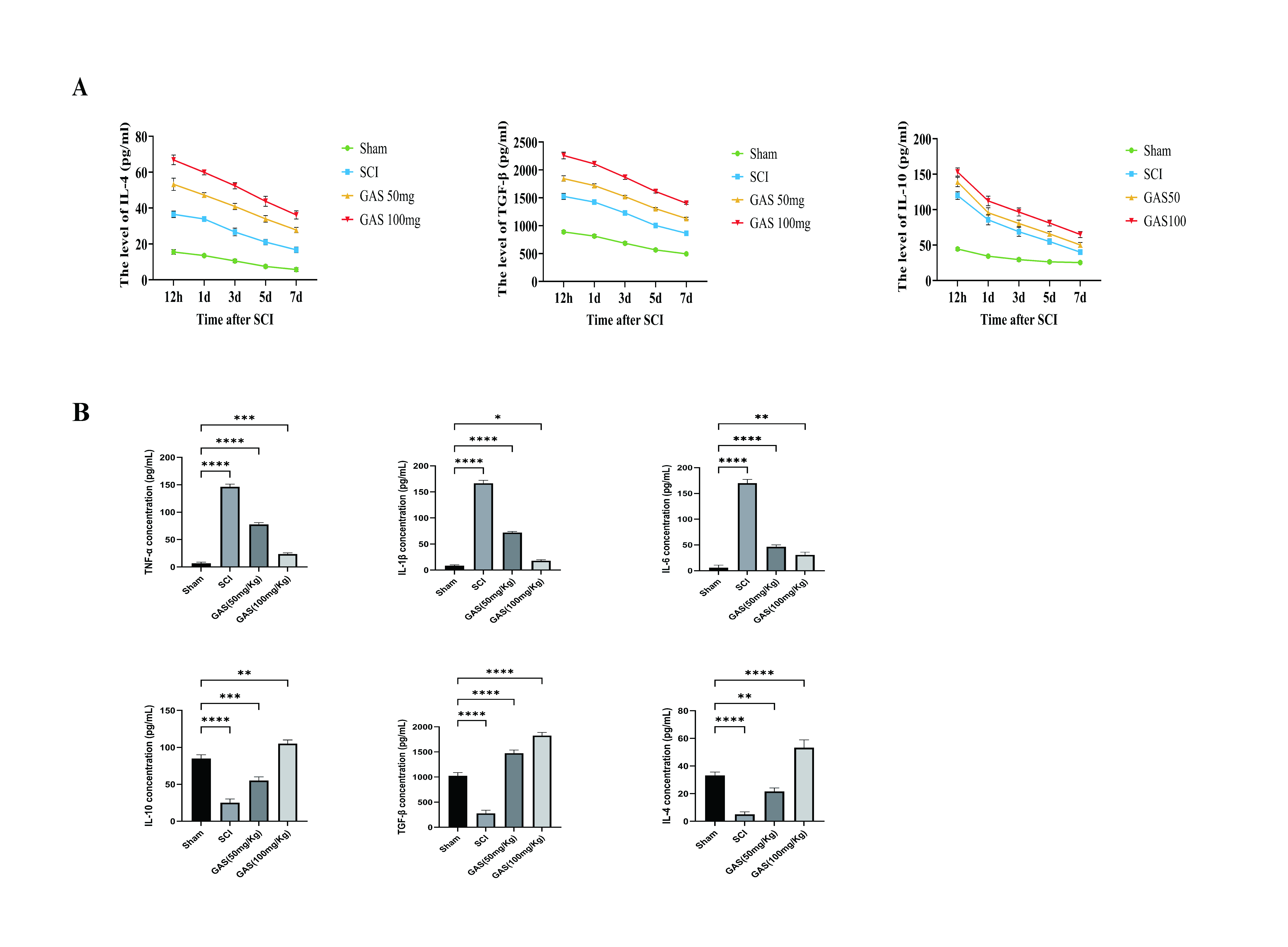

Supplement: Supplementary file 2 — Figure S2: Gastrodin reshapes the cytokine milieu after spinal cord injury. (A) Concentration–time curves of anti‐inflammatory cytokines (IL‐10, TGF‐β, IL‐4) in spinal cord homogenates from different groups of rats at various time points after SCI. (B) ELISA quantification of pro‐ and anti‐inflammatory cytokines (TNF‐α, IL‐1β, IL‐6, IL‐10, TGF‐β, and IL‐4) in spinal cord homogenates from the indicated groups at 12 h, 1 day, 3 days, 5 days, 7 days. [file CNS-32-e70811-s002.jpg]
