# Supplementary material for: The Dual Role of Gastrodin in Spinal Cord Injury: Microglial Phenotype Switching and Neuronal Survival via PI3K/AKT Activation
Source: CNS Neurosci Ther. 2026 Apr 3;32(4):e70811. doi: 10.1002/cns.70811 (PMC13052207; doi:10.1002/cns.70811)
Supplement: Supplementary file 4 — Table S1: Primer sequences and expected amplicon sizes used for mouse RT–qPCR. Primer sequences are listed in the 5′→3′ direction. F and R denote forward and reverse primers, respectively. Product size indicates the expected RT–qPCR amplicon length (bp). Target genes included rat IL‐1β, IL‐6, and TNF‐α; GAPDH was used as the internal reference gene. [file CNS-32-e70811-s001.docx]

| Primers | Sequence (5'→3') | Product Size (bp) |
| --- | --- | --- |
| **IL-1β** | F: TGG ACC TTC CAG GAT GAG GAC A R: GTT CAT CTC GGA GCC TGT AGT G | 84 |
| **IL-6** | F: ACA AGT CGG AGG CTT AAT TAC ACA T R: TTG CCA TTG CAC AAC TCT TTT C | 90 |
| **TNF-α** | F: CCC TCA CAC TCA GAT CAT CTT CT R: GCT ACG ACG TGG GCT ACA G | 61 |
| **GAPDH** | F: AGG TCG GTG TGA ACG GAT TTG R: TGT AGA CCA TGT AGT TGA GGT CA | 123 |
